# NuRepress: Inferring Transcriptional Repressors from Phased Nucleosome Architecture

**DOI:** 10.3390/genes17040480

**Published:** 2026-04-18

**Authors:** Qianming Xiang, Binbin Lai

**Affiliations:** 1 Institute of Medical Technology, Peking University Health Science Center, Beijing 100191, China; 2 Biomedical Engineering Department, Institute of Advanced Clinical Medicine, Peking University, Beijing 100191, China; 3 Department of Dermatology and Venerology, Peking University First Hospital, Beijing 100191, China; 4 State Key Laboratory of Molecular Oncology, Peking University International Cancer Institute, Beijing 100191, China

**Keywords:** transcriptional repressors, repressive chromatin domains, well-phased nucleosome arrays, motif analysis, gene expression variation, epigenomic regulation

## Abstract

Background: The systematic identification of transcriptional repressors remains challenging, as current inference frameworks are predominantly optimized for accessible chromatin, leaving regulatory signals embedded within repressive domains undercharacterized. Methods: Here, we present NuRepress, a computational framework that predicts candidate transcriptional repressors by integrating repressive chromatin architecture, functional signatures, and transcriptional outcomes. NuRepress first identifies well-phased nucleosome arrays within repressive chromatin. These arrays are treated as discrete structural units that capture characteristic local chromatin organization associated with regulatory activity. Since distinct Tn5 cut signal patterns often imply divergent regulatory functions, the framework stratifies these arrays into potential functional subtypes. By synthesizing the quantified repressive efficacy of each subtype with spatial motif enrichment and observed transcriptional dynamics, NuRepress systematically prioritizes and ranks candidate repressors. Results: Our analysis indicated that well-phased nucleosome arrays exhibited accessibility-defined organizational patterns with distinct repressive efficacies, and that these patterns were also observed across species, suggesting that the structural principles captured by NuRepress might extend beyond one specific biological system. Positional motif analysis revealed that distinct TFs exhibited different spatial preferences relative to well-phased nucleosome arrays, suggesting scale-specific preferences for their interactions with these organized chromatin structures. When applied to pancreatic cancer progression, NuRepress identified changes in nucleosome organization associated with stage-specific transcriptional remodeling, highlighting candidate repressors of key oncogenic drivers. Conclusions: NuRepress establishes a structure-aware strategy for repressor inference that extends regulatory genomics beyond accessibility-centered paradigms. By linking well-phased nucleosome organization to transcriptional outcomes, it provides a principled framework for dissecting transcriptional repression across diverse biological settings.

## 1. Introduction

Transcriptional repression forms a fundamental regulatory layer of gene expression, yet its systematic characterization remains less mature than that of activating regulation [1,2]. Many computational frameworks for regulatory inference have been developed around chromatin accessibility and have proven highly effective in delineating active regulatory programs and their associated transcription factors (TFs) [3,4,5]. However, this accessibility-centric lens often overlooks the complex organization of repressive chromatin, where essential regulatory information remains sequestered [6], making the repressors acting in these regions difficult to infer systematically. Consequently, although transcriptional repression is central to cell-state stabilization, developmental restriction, and disease progression [7], the chromatin features that may help illuminate repression-associated regulators remain comparatively underexploited computationally.

Part of this difficulty arises from the fact that repressive regulation is not simply manifested as an absence of activity. Rather, it is frequently embedded within chromatin domains shaped by repressive histone modifications, constrained accessibility, and locally organized nucleosome structure [8,9,10]. From a functional perspective, nucleosome phasing may provide a quantitative, tractable architectural readout that brings these diverse chromatin signals into a more coherent regulatory context. Well-phased nucleosome arrays are recurrently observed in regulatory regions and can reflect local chromatin architectures associated with regulatory activity [11,12]. Within repressive chromatin domains, however, the precise regulatory grammar linking nucleosome phasing to repressor recruitment remains elusive. In some cases, ordered nucleosome organization may arise downstream of TF binding or chromatin remodeling; in others, it may provide a structural setting that facilitates or stabilizes repression-associated interactions [13,14]. Although the causal direction of this association is not yet fully resolved, these observations raise the possibility that the phasing status of nucleosomes within repressive domains may encode interpretable information relevant to transcriptional regulation.

Recent studies have substantially expanded the identification and functional characterization of repressive regulatory elements. Huang et al. identified human silencers by correlating cross-tissue epigenetic profiles with gene expression and further used sequence features to predict additional silencers [15]. Jayavelu et al. developed a subtractive framework to define uncharacterized cis-regulatory elements, functionally tested ~7500 candidates by MPRA, and then trained an SVM classifier to predict candidate silencers across human and mouse cell and tissue types [16]. Pang and Snyder established the ReSE screen to systematically identify silencer regions in human cells on the basis of silencer-mediated repression of an inducible cell-death reporter [17]. More recently, SilenceREIN incorporated chromatin loop-anchor information through a graph neural network to seek silencers within regulatory interaction networks [18], whereas MPRAduo-based analyses of RE1/REST elements provided a high-resolution, family-specific view of one major silencer class [19]. Collectively, these approaches have broadened the catalog of repressive elements, yet an important layer of information within repressive chromatin remains insufficiently explored. Repressive histone-modification profiles are generated from nucleosome-associated DNA and therefore encode both domain-level repression signals and local nucleosome phasing information—a duality not yet systematically leveraged for repression-oriented regulatory inference.

Here, we present NuRepress, a computational framework that predicts candidate transcriptional repressors by integrating repressive chromatin architecture, functional signatures, and transcriptional outcomes. NuRepress first identifies well-phased nucleosome arrays within repressive chromatin and treats them as discrete structural units that capture characteristic local chromatin organization associated with regulatory activity. Phased nucleosome arrays are not exclusive to repressive chromatin; in this study, we analyze such organization specifically within repressive chromatin domains, where structural phasing and repression-associated context can be considered jointly. It then stratifies these arrays into potential functional subtypes based on distinct Tn5 cut signal patterns and synthesizes subtype-specific repressive efficacy, spatial motif enrichment, and observed transcriptional dynamics to prioritize candidate repressors. We show that well-phased nucleosome arrays in repressive chromatin exhibit accessibility-defined organizational patterns with distinct repressive efficacies, and that these patterns are also observed across species, suggesting that the structural principles captured by NuRepress may extend beyond one specific biological system. We further show that distinct TFs display different spatial preferences relative to well-phased nucleosome arrays, pointing to scale-specific modes of interaction with these organized chromatin structures. When applied to pancreatic cancer progression, NuRepress identifies changes in nucleosome organization associated with stage-specific transcriptional remodeling, highlighting candidate repressors of key oncogenic drivers. Taken together, these findings establish phased nucleosome architecture as an informative substrate for repressor inference and position NuRepress as a complementary framework for interrogating transcriptional repression beyond accessibility-centered paradigms.

## 2. Materials and Methods

### 2.1. Overall Design of NuRepress

**Figure 1:**
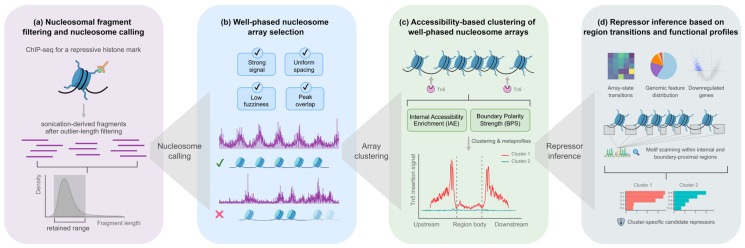
Overview of the NuRepress framework. (**a**) Repressive domains are defined from ChIP-seq signals of repressive histone marks. (**b**) Well-phased nucleosome arrays are selected as structural units. Nucleosome shading indicates positioning confidence, with darker colors denoting higher confidence. (**c**) ATAC-seq-derived accessibility features are used to classify arrays into distinct accessibility-defined subtypes. (**d**) Distance-resolved motif analysis and transcriptional information are integrated with subtype-specific repressive regulatory potential to prioritize cluster-specific candidate repressors.

NuRepress is a modular computational framework designed for the systematic inference of candidate transcriptional repressors from repressive chromatin domains. NuRepress first delineates repressive chromatin domains based on ChIP-seq signals of repressive histone marks and infers nucleosome positioning patterns within these regions (Figure 1a). Well-phased nucleosome arrays are then identified and treated as discrete structural units representing local chromatin organization (Figure 1b). For each array, chromatin accessibility features derived from ATAC-seq signals are quantified to characterize the accessibility landscape surrounding the array and to stratify arrays into distinct accessibility-defined subtypes (Figure 1c). In parallel, motif occurrences are profiled in both internal and boundary-proximal regions of nucleosome arrays using distance-resolved scanning. Finally, subtype-specific repressive regulatory potential, spatial motif enrichment patterns, and associated transcriptional outcomes are integrated to prioritize TFs with potential repressive activity (Figure 1d).

NuRepress requires three primary categories of input data: ChIP-seq data of a repressive histone modification, chromatin accessibility data (ATAC-seq), and gene expression profiles. Together, these datasets enable a stepwise analysis in which the relationship between repressive chromatin architecture and transcriptional regulation is progressively delineated, thereby supporting candidate repressor prioritization within an explicitly defined structural context.

### 2.2. Identification of Well-Phased Nucleosome Arrays

Repressive histone mark ChIP-seq data were first subjected to fragment-length outlier filtering. Extreme fragment-length outliers were removed using median absolute deviation–based thresholds, in order to reduce technical noise while preserving the intrinsic fragment-length variability present in ChIP-seq data [20]. Genome-wide nucleosome calling was performed using DANPOS [21], and the resulting nucleosome positions were characterized in terms of occupancy signal strength, positional fuzziness, and inter-nucleosome spacing. For the identification of well-phased nucleosome arrays, NuRepress implemented two algorithms—a sliding-window merging approach and a seed-and-extend approach—which could be selected according to the expected structural regularity of the target chromatin context.

The sliding-window merging approach evaluated consecutive windows of *k* nucleosomes (default: 3) against predefined phasing criteria, merged passing windows that overlapped or fell within a permissible gap (default: 200 bp), and then applied a second round of array-level filtering to the merged regions, as locally compliant windows did not necessarily retain their phasing properties at the array scale. The seed-and-extend approach identified short high-confidence seed arrays of *k* nucleosomes as structural anchors, then extended each seed iteratively by adding one nucleosome at a time to the side that yielded the greater improvement in overall array quality under slightly relaxed thresholds (1.2× those used for seed selection), with extension on each side terminated upon reaching a maximum consecutive failure limit (default: 2).

In both strategies, candidate arrays were filtered using the following default criteria. First, inter-nucleosome spacing was required to fall within 160–220 bp with a coefficient of variation below 0.15. This range accommodated canonical nucleosome repeat lengths by allowing variation in linker DNA together with the nucleosomal DNA occupied by adjacent nucleosomes. Second, mean and minimum signal intensities were required to exceed the 30th and 20th percentiles of the genome-wide nucleosome signal distribution, respectively. Third, positional fuzziness was required to remain below the 20th percentile of the genome-wide fuzziness distribution, thereby retaining only well-positioned nucleosomes. In addition, filtered arrays were required, by default, to overlap enrichment peaks by at least 30% of their length. Finally, arrays passing all criteria were deduplicated and merged by genomic coordinates to generate the well-phased nucleosome arrays that served as the structural basis for downstream analysis.

### 2.3. Accessibility Profiling and Unsupervised Subtyping of Well-Phased Nucleosome Arrays

To identify functionally distinct subtypes of well-phased nucleosome arrays on the basis of their chromatin accessibility profiles, NuRepress integrated ATAC-seq Tn5 insertion signals, which were first normalized to counts per million (CPM) to correct for sequencing depth variation across samples. Since nucleosome arrays varied in length, each array body was uniformly divided into *n* equal-length bins (default: 100) to enable signal aggregation under a standardized relative coordinate system. In addition, the left and right boundaries of each array were extended by 1000 bp by default to capture accessibility patterns in the flanking chromatin environment.

Two quantitative measures were derived for each array *i*. The Internal Accessibility Enrichment (IAE) quantified the relative openness of the array interior with respect to its flanking regions:

(1) $${IAE}_{i}={log}_{2}\frac{{ATAC\_mean}_{inside}\left(i\right)+\varepsilon }{{[ATAC\_mean}_{left}(i)+{ATAC\_mean}_{right}(i)]/2+\varepsilon }$$

where ${ATAC\_mean}_{inside}\left(i\right)$ denotes the mean Tn5 insertion signal within the array interior, ${ATAC\_mean}_{left}(i)$ and ${ATAC\_mean}_{right}(i)$ denote the mean signals in the left and right flanking regions, respectively, ε is a small pseudocount (default: 10^−9^) to avoid division by zero. Negative IAE values indicate a more closed interior relative to the flanking regions. The Boundary Polarity Strength (BPS) quantified the degree of asymmetry between the left and right flanking accessibility signals:

(2) $${BPS}_{i}=\left|{\;log}_{2}\frac{{ATAC\_mean}_{right}\left(i\right)+\varepsilon }{{ATAC\_mean}_{left}\left(i\right)+\varepsilon }\;\right|$$

Larger BPS values reflect greater left–right asymmetry in the boundary chromatin environment, whereas values approaching zero indicate comparable accessibility on both sides. Together, IAE and BPS mapped each array into a two-dimensional accessibility feature space.

To improve comparability across samples, features were standardized using within-sample z-score normalization prior to clustering. Unsupervised clustering was then performed using a Gaussian mixture model implemented in mclust [22], with the optimal number of components selected automatically by the Bayesian Information Criterion. Clustering quality was assessed using the mean silhouette coefficient $\bar{s}$ across all arrays:

(3) $$\left\{\begin{array}{c} \bar{s}=\frac{1}{n}\sum_{i=1}^{n}s(i)\\ s\left(i\right)=\frac{b\left(i\right)-a\left(i\right)}{\max\left\{a\left(i\right),b\left(i\right)\right\}} \end{array}\right.$$

Here, $a\left(i\right)$ denotes the mean intra-cluster distance for array *i*, $b\left(i\right)$ denotes the minimum mean distance from array *i* to any other cluster, and *n* denotes the total number of arrays, all computed in the IAE–BPS feature space.

### 2.4. State Transition Analysis and Genomic Annotation of Well-Phased Nucleosome Arrays

To enable cross-sample comparison, arrays from the same chromosome overlapping by at least 50% (default) were merged into union regions, accommodating minor boundary variation while preserving structural correspondence. For each union region, the presence or absence of an array and its accessibility subtype were jointly encoded as a unified state variable, defining the state space as $S=\left\{None,C_{1},C_{2},\ldots ,C_{k}\right\}$ for *k* clusters, where *None* denotes the absence of a well-phased array. For any two biologically ordered samples or conditions *A* and *B*, a (k+1)×(k+1) transition matrix *T* was constructed, in which rows represent the initial state in sample *A*, columns represented the final state in sample *B*, and each element $T_{i,j}$ recorded the number of union regions transitioning from state *i* to state *j*.

In order to link structural state transitions to genomic context, each array was functionally annotated and compared across accessibility subtypes for enrichment in genomic elements. Given the central role of promoters as integration hubs for transcriptional regulatory signals, a binary promoter annotation matrix *Q* was defined, in which element $Q_{i,g}=1$ if array *i* overlapped the promoter of gene g, and $Q_{i,g}=0$ for non-overlapping cases.

### 2.5. Repressor Inference Through Repressive Efficacy-Weighted Motif Enrichment

For two samples *A* and *B*, the ${log}_{2}$ fold change in expression for gene g was denoted $e_{g}^{A\to B}$. A binary downregulation indicator vector $d^{A\to B}$ was defined to indicate genes downregulated in *B* relative to *A*.

For any transition type *i* → *j* of interest identified from *T*, a binary indicator vector $x_{i\to j}^{A\to B}$ was defined over all union regions, with $x_{k,i\to j}^{A\to B}=1$ if region *k* transitioned from state *i* to state *j*, and 0 otherwise. The structural-expression association matrix was then computed as $C_{i\to j}^{A\to B}={\left(x_{i\to j}^{A\to B}\right)}^{T}Q$, yielding a row vector over genes that records the number of transitioning arrays associated with each gene’s promoter. The optional foreground array set $F_{i\to j}^{A\to B}$ for downstream motif analysis was defined as:

(4) $$F_{i\to j}^{A\to B}=\{array\;k:\;x_{k,\;i\to j}^{A\to B}=1\;and\;\exists g\;\;s.t.\;\;Q_{k,g}=1\;and\;d_{g}^{A\to B}=1\}$$

When the proportion of well-phased arrays overlapping promoters exceeded a user-specified threshold (default: 20%), $F_{i\to j}^{A\to B}$ was used as the foreground for motif enrichment; otherwise, all arrays of the corresponding subtype were used to preserve statistical power. Motif enrichment analysis was performed using HOMER [23] on linker DNA within well-phased arrays and on 200 bp windows extending up to 1000 bp (default) from the phasing boundary, defined as the array edge exhibiting the steeper decline in nucleosome occupancy signal, which was used as an operational reference point to capture the more clearly resolved transition between the phased array and the surrounding chromatin context [24,25]. HOMER’s default GC content-matched background was used, with optional stricter constraints such as matching by genomic context and array accessibility subtype.

To quantify the repressive relevance of each accessibility subtype, a Repressive Efficacy score (${RE}_{s,c}$) was defined for subtype *C* in sample *S*. Drawing on *Q*, genes were grouped into those whose promoters contained only subtype *C* arrays ($G_{s,c}^{only}$) or contained no well-phased arrays at all ($G_{s}^{absent}$), and the effect size was computed as:

(5) $$\Delta_{s,c}=median\left(E_{s,g}\;|\;g\in G_{s,c}^{only}\right)-median\left(E_{s,g}\;|\;g\in G_{s}^{absent}\right)$$

where $E_{s,g}$ denotes the expression level of gene g in sample *S*. A one-sided Wilcoxon rank-sum test (alternative hypothesis: $G_{s,c}^{only}$ < $G_{s}^{absent}$) was applied, with the resulting *p*-value denoted $P_{s,c}^{expr}$. The Repressive Efficacy score was then defined as:

(6) $${RE}_{s,c}=\left[\Delta_{s,c}<0\right]\cdot |\Delta_{s,c}|\cdot \;-lg\;(P_{s,c}^{expr})$$

where $\left[\Delta_{s,c}<0\right]$ is an indicator function that takes the value 1 only when $\Delta_{s,c}$ is negative, ensuring that ${RE}_{s,c}$ is non-zero only when subtype *C* is associated with gene downregulation ${RE}_{s,c}$ = 0 indicates no evidence of repression; larger values reflect stronger association with downregulation.

The Motif Potential (${MP}_{s,c,t}$) for TF *t* was defined as the maximum enrichment significance (adjusted) across all boundary windows *b*, and the Repressor Prediction Score (${RPS}_{s,c,t}$) for TF *t* in subtype *C* of sample *S* was defined as:

(7) $$\left\{\begin{array}{c} {RPS}_{s,c,t}={RE}_{s,c}\cdot \;{MP}_{s,c,t}\\ {MP}_{s,c,t}=\max\;(\left[-lg(P_{s,c,t,b}^{motif})\right]) \end{array}\right.$$

A TF *t* was considered a candidate repressor for subtype *C* only when ${RE}_{s,c}$ > 0 and ${MP}_{s,c,t}>-lg(\alpha )$, where α denotes the user-specified significance threshold (default: 0.05). To characterize subtype preference for each candidate repressor, a Specificity Score (*S_score*) was additionally defined as:

(8) $${S\_score}_{s,c,t}={MP}_{s,c,t}-{max}_{c^{\prime }\neq c}\;{MP}_{s,c^{\prime },t}$$

The Specificity Score reflects the relative enrichment evidence for TF *t* in subtype *C* compared to all other subtypes, and is retained as a descriptive metric characterizing subtype preference rather than a mandatory filtering criterion, with values close to zero in absolute value indicating limited subtype bias and therefore requiring particularly careful interpretation. All results shown in the subsequent analyses were generated using the default settings.

### 2.6. Data Overview

To evaluate the applicability of NuRepress in a biomedical research context, multi-omics data from human pancreatic cell lines were analyzed, encompassing ChIP-seq, ATAC-seq, and RNA-seq profiles across three cell types: normal human pancreatic epithelial cells (HPNE), primary pancreatic cancer cells (PANC-1), and metastatic pancreatic cancer cells (Capan-1), aligned to the hg19 reference genome. To further validate the cross-species generalizability of primary observations, mouse Patski cell line data were additionally incorporated. The following datasets were used:

- H3K27me3 ChIP-seq: hg19, HPNE, PANC-1, Capan-1, GSE149103 (GEO); mm10, Patski, ENCSR942RCG (ENCODE)
- ATAC-seq: hg19, HPNE, PANC-1, Capan-1, GSE149103 (GEO); mm10, Patski, ENCSR351QUO (ENCODE)
- RNA-seq: hg19, HPNE, PANC-1, Capan-1, GSE149103 (GEO); mm10, Patski, GSE59779 (GEO)

## 3. Results

### 3.1. Well-Phased Nucleosome Arrays Identified by Nurepress Demonstrate Interpretability for Transcriptional Regulatory Analysis

To illustrate the utility of NuRepress in biomedical research, three pancreatic cell lines with a defined disease progression relationship—normal human pancreatic epithelial cells (HPNE), primary pancreatic cancer cells (PANC-1), and metastatic pancreatic cancer cells (Capan-1)—were used as input data. Starting from genome-wide nucleosome predictions, NuRepress applied the seed-and-extend algorithm by default to identify well-phased nucleosome arrays within H3K27me3-marked repressive domains.

**Figure 2:**
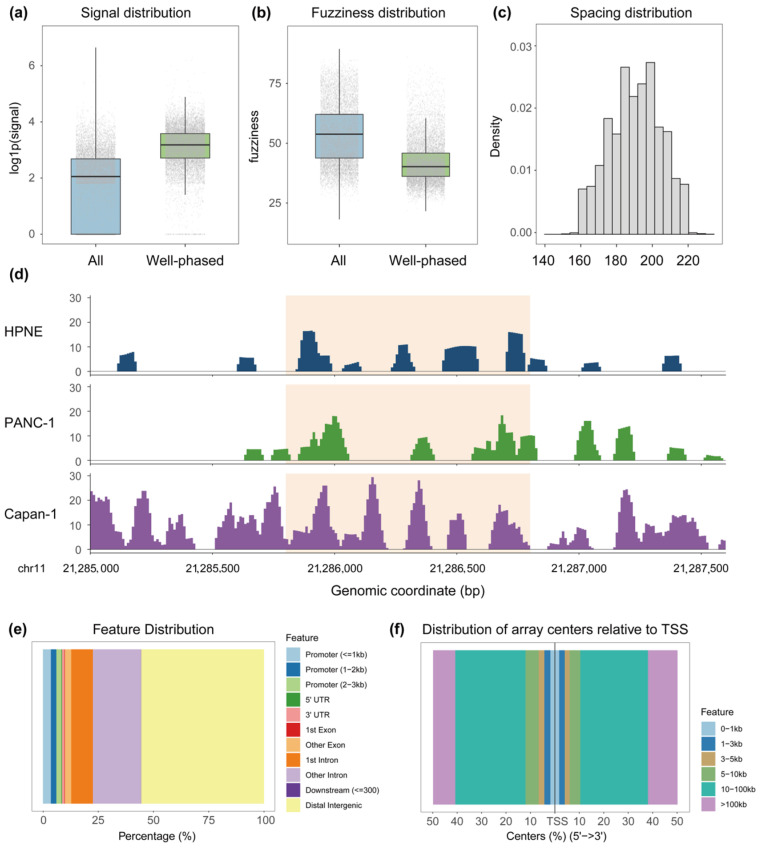
Structural characterization and genomic annotation of well-phased nucleosome arrays identified by NuRepress. (**a**) Comparison of nucleosome occupancy signal between array-internal nucleosomes and all predicted nucleosomes. (**b**) Comparison of positional fuzziness between array-internal nucleosomes and all predicted nucleosomes. (**c**) Inter-nucleosome spacing distribution within well-phased arrays. (**d**) A representative genomic region in which well-phased nucleosome arrays were identified in Capan-1 cells but not in HPNE or PANC-1 cells. The y-axis represents the nucleosome prediction signal output by DANPOS. (**e**) Genomic functional annotation of all well-phased arrays. (**f**) Distribution of all well-phased arrays relative to the nearest transcription start site (TSS).

Compared to all predicted nucleosomes, those retained within well-phased arrays exhibited higher occupancy signal strength (Figure 2a), lower positional fuzziness (Figure 2b), and inter-nucleosome spacing consistent with canonical nucleosome repeat length (Figure 2c). Arrays were further filtered by repressive domain overlap to ensure structural analysis was conducted within a repressive chromatin context (Figure 2d).

Genomic annotation revealed that well-phased arrays were predominantly distributed in distal intergenic and intronic regions, with a relatively lower proportion at promoter-proximate regions (Figure 2e–f), consistent with the broad domain organization characteristic of H3K27me3-mediated Polycomb repression [26]. This does not preclude a role for such arrays near promoters, as H3K27me3 can also form focal enrichment at TSS-proximate regions in the context of Polycomb-mediated promoter silencing and bivalent chromatin states [8].

Together, these results suggest that the well-phased arrays identified by NuRepress represent structurally coherent chromatin units within a repressive context and support their use as a defined substrate for downstream repressor inference.

### 3.2. Well-Phased Nucleosome Arrays Display Generalizable Accessibility-Based Subtypes with Distinct Biological Signatures

**Figure 3:**
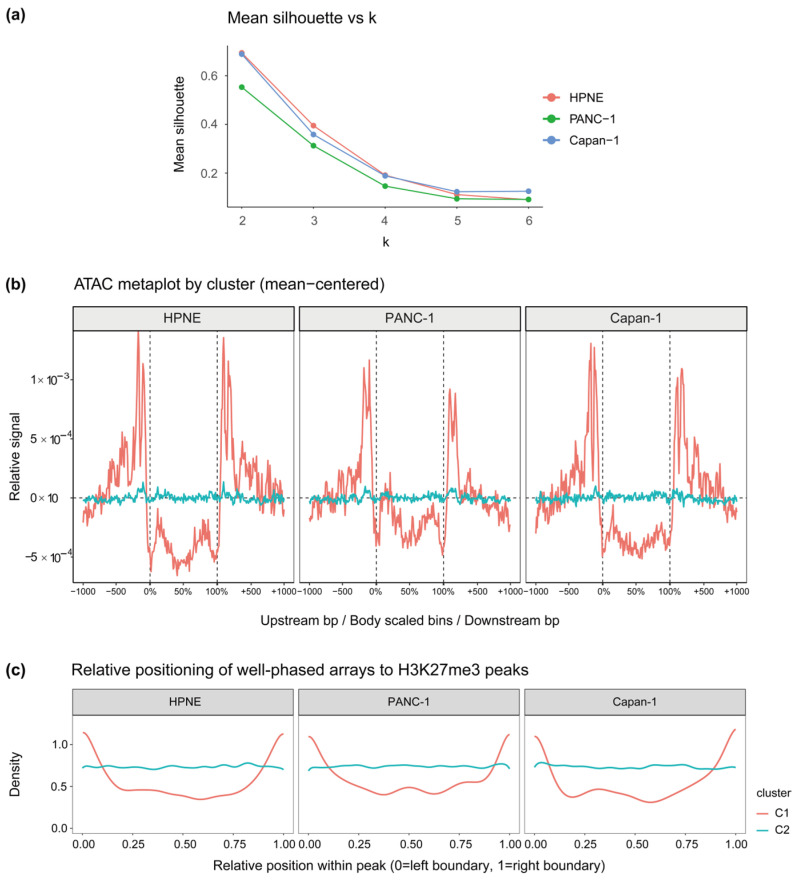
Accessibility-based subtyping of well-phased nucleosome arrays identified by NuRepress reveals concordant patterns across samples. (**a**) Mean silhouette coefficients for accessibility-based subtyping at varying values of *k* across samples, used to determine the optimal number of subtypes. (**b**) Aggregate Tn5 insertion signal profiles for each subtype across samples, centered by within-cluster mean. (**c**) Relative positional distribution of each accessibility subtype within repressive histone mark enrichment peaks across samples, with 0 and 1 denoting the two boundaries of each peak region.

Well-phased nucleosome arrays identified across all three human cell lines (HPNE, PANC-1, and Capan-1) were further stratified by their chromatin accessibility profiles using IAE and BPS computed from ATAC-seq Tn5 insertion signals. Rather than requiring a user-specified number of clusters, NuRepress automatically determined the optimal *k* by maximizing the mean silhouette coefficient over a predefined search range, identifying *k* = 2 as the optimal solution in all three samples (Figure 3a).

The two subtypes exhibited inverse yet consistent Tn5 insertion profiles across all three cell lines (Figure 3b): C1 arrays displayed a pronounced accessibility gradient at array boundaries, with relatively lower accessibility in the interior and higher accessibility in the flanking regions; C2 arrays, by contrast, showed a uniformly distributed accessibility signal with no discernible gradient at boundaries. To further confirm that these subtype distinctions reflect genuine chromatin accessibility differences rather than sequence composition biases intrinsic to Tn5 insertion preference, array length and GC content were compared across subtypes within each sample (Figure S1a,b). No statistically significant differences were observed in either metric (Kruskal–Wallis test, *p* > 0.05 in all cases). Together, the concordance of these profiles across independent samples suggested that NuRepress could capture shared accessibility patterns of well-phased nucleosome arrays in distinct biological settings.

The biological relevance of this subtyping scheme was further supported by the distinct positional preferences of the two subtypes: C1 arrays were preferentially enriched near repressive domain boundaries, whereas C2 arrays were uniformly distributed throughout domain interiors (Figure 3c). This positional distinction aligned with their respective accessibility profiles—the sharp boundary gradient of C1 may reflect a role in chromatin domain insulation, while the uniform signal of C2 was suggestive of a role in maintaining nucleosome organization within the repressive domain interior. These patterns suggested that the accessibility-based subtyping framework implemented by NuRepress may delineate functionally distinct array populations, with chromatin accessibility profiles serving as a putative reflection of differential regulatory potential.

Beyond sample-level generalization, NuRepress was further applied to mouse Patski cell data to assess cross-species applicability. The same two-subtype solution was identified as optimal (Figure S2a), and both the accessibility profiles (Figure S2b) and domain-relative positional distributions (Figure S2c) closely recapitulated the patterns observed in human samples, suggesting that the subtyping structure identified by NuRepress may reflect a shared organizational principle across species. Based on their characteristic accessibility and positional profiles, we hereafter refer to C1 and C2 as boundary-transition arrays and accessibility-uniform arrays, respectively.

Taken together, these results indicate that NuRepress resolves well-phased nucleosome arrays into accessibility-based subtypes with consistent structural, positional, and putative functional characteristics across samples and species, providing a biologically interpretable foundation for downstream repressor inference.

### 3.3. NuRepress Quantifies Subtype-Specific Repressive Efficacy and Prioritizes Candidate Transcriptional Repressors

**Figure 4:**
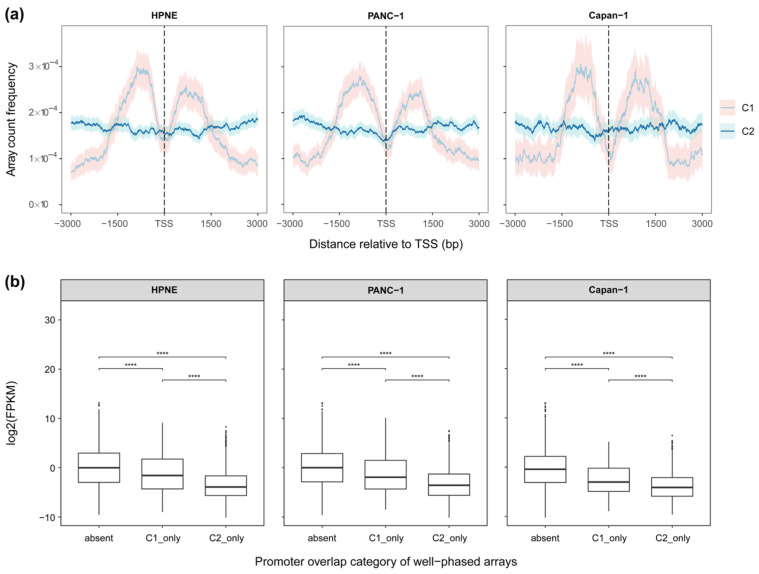
Distinct effects of different nucleosome array subtypes on gene expression. (**a**) Positional frequency distribution of different array subtypes around the TSS across samples. The solid line represents the expected value, and the shaded area indicates the 95% confidence interval (bootstrap, 500 resamples). (**b**) Comparison of overall gene expression levels across samples among genes whose promoter regions contain no well-phased arrays ($G^{absent}$), only C1 arrays ($G_{C1}^{only}$), or only C2 arrays ($G_{C2}^{only}$). Asterisks denote BH-adjusted significance levels from pairwise Wilcoxon tests: **** *p*.adj ≤ 0.0001.

Having established accessibility-based subtypes with distinct structural and positional characteristics, NuRepress further evaluated the association between subtype identity and transcriptional regulation. The TSS-proximal positional distribution of boundary-transition (C1) and accessibility-uniform (C2) arrays was first compared across samples (Figure 4a). Boundary-transition arrays exhibited a more pronounced peak-valley structure flanking the TSS, with lower density immediately around the TSS and elevated density on both sides, potentially consistent with a role in promoter-proximal boundary organization and local chromatin compartmentalization. Accessibility-uniform arrays displayed a shallower and more uniform gradient in the same region, suggestive of a more diffuse repressive organization within domain interiors.

To assess the functional relevance of each subtype, genes were grouped into those whose promoters harbored exclusively C1 arrays ($G_{C1}^{only}$), exclusively C2 arrays ($G_{C2}^{only}$), or no well-phased arrays ($G^{absent}$). Both $G_{C1}^{only}$ and $G_{C2}^{only}$ exhibited significantly lower expression than $G^{absent}$ (*p* < 0.01; Figure 4b), with $G_{C2}^{only}$ showing a larger magnitude of downregulation. An analogous pattern of differential gene downregulation between the two subtypes was also observed in mouse Patski cells (Figure S2d). NuRepress quantified the Repressive Efficacy (RE) of each subtype accordingly (Table 1 and Table S1), yielding RE > 0 for both subtypes across all samples, confirming their association with transcriptional repression. The substantially higher RE values of accessibility-uniform arrays relative to boundary-transition arrays suggest that the former were more strongly associated with active repressive regulation, whereas the lower RE values of the latter might primarily reflect structural constraints related to promoter-proximal chromatin boundaries. Collectively, these results supported the ability of NuRepress to distinguish array subtypes with different levels of repressive relevance.

**Figure 5:**
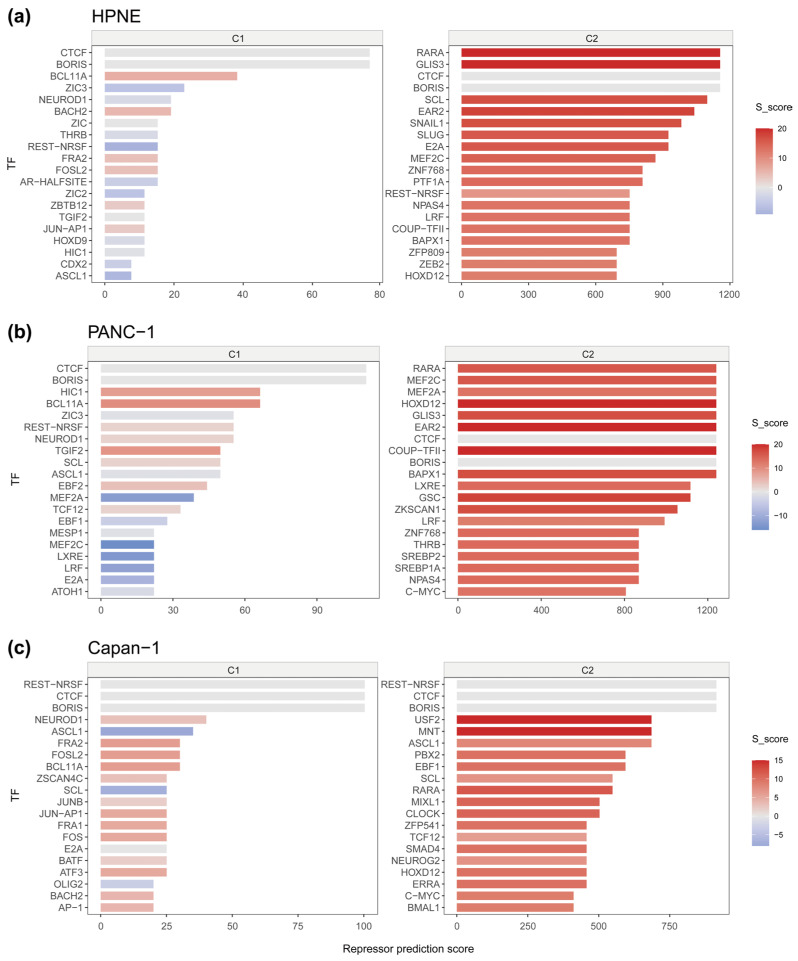
Repressors inferred by NuRepress across samples (top 20 TFs for each array subtype). (**a**–**c**) correspond to the HPNE, PANC-1, and Capan-1 samples, respectively. The x-axis represents the repressor prediction score (RPS) of each TF within each subtype. Bar colors indicate the Specificity Score (S_score). Absolute S_score values closer to zero indicate a higher degree of shared enrichment across subtypes.

Building on these RE estimates, NuRepress performed motif enrichment analysis on linker DNA regions and boundary-proximate windows of well-phased arrays, and integrated the resulting Motif Potential (MP) scores with subtype-level RE values to compute Repressor Prediction Scores (RPSs) for candidate repressors in each sample. Candidate repressors associated with accessibility-uniform arrays consistently yielded higher RPS values than those associated with boundary-transition arrays across all samples (Figure 5a–c), reflecting the stronger repressive efficacy of C2 and suggesting greater transcriptional repression potential for C2-associated candidates.

Among the top-ranked predictions, CTCF/BORIS family members were consistently identified in both subtypes across all samples. Notably, their Specificity Scores (S_score) remained close to zero, indicating similar enrichment across subtypes rather than a clear subtype-biased association. This pattern suggests that high ranking alone does not necessarily imply subtype specificity. Considering the established functions of CTCF and BORIS in chromatin boundary insulation and nucleosome organization [27,28,29,30], their recurrent detection across subtypes may more plausibly reflect a constitutive role in structural maintenance of phased chromatin architecture.

In contrast, REST/NRSF in the Capan-1 sample also displayed high RPS values in both subtypes together with an S_score near zero, but its biological interpretation differs. Because REST/NRSF is a well-characterized transcriptional repressor that promotes gene silencing through recruitment of repressive chromatin machinery, and has also been implicated in endocrine differentiation during pancreatic organogenesis [31], its shared enrichment across subtypes may indicate broadly deployed repressive activity rather than merely structural maintenance. A position-resolved comparison further suggests that CTCF/BORIS and REST/NRSF may differ in their local enrichment patterns around well-phased array boundaries, which may in turn be consistent with their inferred functional tendencies [32] (Figure S3).

Thus, the comparison between CTCF/BORIS and REST/NRSF illustrates that similar S_score patterns might correspond to distinct biological meanings. Within NuRepress, S_score therefore provides an important interpretive dimension by separating subtype-specific candidates from subtype-shared factors, while also enabling the functional discrimination between components likely involved in structural maintenance and those likely engaged in active repressive regulation.

To assess the added value of the phasing-based array step beyond repressive domain context alone, we repeated the downstream NuRepress workflow using H3K27me3-enriched domains as input regions. The optimal clustering solution remained k = 2, indicating that a coarse accessibility-based dichotomy was still detectable at the domain level (Figure S4). However, compared with well-phased nucleosome arrays, the resulting subtype patterns were less homogeneous, with C1 showing clear intra-region heterogeneity, especially in HPNE, and milder broadening also evident in PANC-1 and Capan-1. At the level of repressor prediction, the domain-based analysis retained only partial overlap with the array-based results and, across all three samples, was generally less effective in recovering candidates with clearer literature support for transcriptional repression (Figure S5). Most notably, the RARA-centered signal, which was consistently retained in the array-based analysis across HPNE, PANC-1, and Capan-1, was lost in the domain-based results. In parallel, candidates with established repressive relevance, including SNAIL1, SLUG, LRF, and HIC1, were also weakened or no longer distinctly recovered, while the predictions shifted toward broader or less clearly repressor-associated factors. These comparisons suggest that the phasing-based array step does not merely refine domain-level patterns, but improves the recovery of candidate repressors with stronger functional grounding.

As an additional comparative analysis, we also applied diffTF [2] to the same ATAC-seq and RNA-seq datasets from HPNE, PANC-1, and Capan-1. Across pairwise comparisons, diffTF identified 45 candidate repressors (adjusted *p*-value < 0.05 in at least one comparison), among which five, namely RARA [33], SNAI2/SLUG [34], MESP1 [35], MNT [36], and ZIC2 [37], were also included among the top 15 repressors predicted by NuRepress in at least one of the three samples (Figure 5 and Figure S6). Although the overlap was limited in absolute number, the shared candidates are nevertheless notable, as they include factors with reported or plausible repressive relevance. It is also noteworthy that a subset of candidates uniquely prioritized by NuRepress, including BACH2 [38], BCL11A [39], HIC1 [40], TGIF2 [41], SNAIL1 [42], and LRF [43], have prior literature support for transcriptional repressive functions, with some additionally implicated in pancreatic cancer progression, yet were not identified by diffTF in the present analysis. Taken together, these findings suggest that the concordance between diffTF and NuRepress, although limited, is still informative, while the additional recovery by NuRepress of literature-supported repressive candidates may provide further support for the reliability of the NuRepress framework.

Taken together, these results suggest that, although repressive chromatin domains alone retain part of the relevant biological signal, the incorporation of phased nucleosome architecture enables NuRepress to define more structurally coherent analytical units and to derive more focused repressor predictions with clearer biological interpretability.

### 3.4. NuRepress Reveals Repressor-Associated Nucleosome Reorganization During Pancreatic Cancer Progression

To evaluate whether NuRepress can capture biologically interpretable regulatory signals in a disease-relevant context, we applied it to samples representing three stages of pancreatic cancer progression—normal pancreatic epithelial cells (HPNE), primary pancreatic cancer cells (PANC-1), and metastatic pancreatic cancer cells (Capan-1)—and interrogated the predicted repressors against established disease biology.

Several top-ranked candidates across samples have documented associations with pancreatic cancer (Figure 5a–c). RARA (RARα), which attained the highest RPS in both HPNE and PANC-1, recruits co-repressor complexes including SMRT/N-CoR and histone deacetylases in its unliganded state [33], and higher RARα expression has been associated with improved overall survival [44]. HIC1 is frequently inactivated by promoter hypermethylation in pancreatic cancer, with low expression correlated with abbreviated survival [45], and has been shown to suppress IL-6/STAT3 signaling and downstream effectors including c-Myc and MMP family members [40]. TGIF2, by contrast, has been implicated as a pro-malignant factor promoting epithelial–mesenchymal transition (EMT) and hepatic metastasis through cooperation with Smad2 and potentiation of EGFR/MAPK signaling [46]. Further predicted candidates including SNAI1 [47], SLUG [48], LRF [39], and MNT [49] carry documented roles in EMT, transcriptional repression, and oncogenic network regulation.

**Figure 6:**
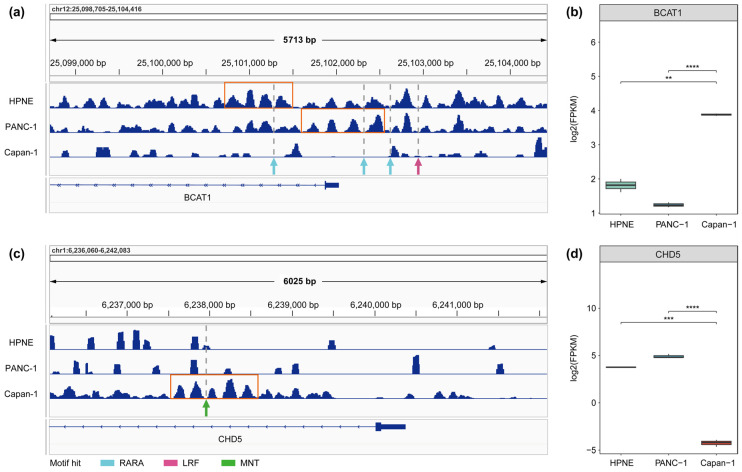
NuRepress identifies and interprets repressive chromatin contexts associated with key genes during pancreatic cancer progression. Genome browser tracks show nucleosome signals derived from H3K27me3 ChIP-seq data processed by DANPOS. (**a**) Well-phased repressive nucleosome arrays are present at the BCAT1 promoter in HPNE and PANC-1 (orange boxes), but absent in Capan-1. (**b**) Expression levels of BCAT1 across samples. (**c**) Well-phased repressive nucleosome arrays at the CHD5 promoter are detected only in Capan-1 (orange boxes), whereas HPNE and PANC-1 show sparse repressive signals without such arrays. (**d**) Expression levels of CHD5 across samples. Arrows indicate genomic positions of motif matches with high repressor prediction scores (RPS). Asterisks denote BH-adjusted significance levels from pairwise Wilcoxon tests: ** *p*.adj ≤ 0.01, *** *p*.adj ≤ 0.001, and **** *p*.adj ≤ 0.0001.

Along the progression from normal pancreatic epithelial cells (HPNE) through primary pancreatic cancer cells (PANC-1) to metastatic pancreatic cancer cells (Capan-1), stage-specific gene expression shifts underlie cellular state transitions. Among the genes implicated in this process, BCAT1 and CHD5 represent well-characterized examples of pro-tumorigenic and tumor-suppressive functions, respectively. BCAT1, a pro-metastatic determinant whose elevated expression is robustly associated with adverse prognosis [49], drives tumor progression through branched-chain amino acid catabolism, mTOR pathway activation, and neutrophil recruitment into the tumor microenvironment [50,51]. CHD5, by contrast, is a bona fide tumor suppressor that constrains aberrant proliferation through maintenance of the Cdkn2a regulatory network [52] and negative regulation of the G2/M checkpoint kinase WEE1 [53]; its loss of expression is a recognized indicator of poor clinical outcomes in pancreatic cancer patients [54]. Consistent with these functional profiles, BCAT1 expression was significantly lower in HPNE and PANC-1 than in Capan-1 (Figure 6b), while CHD5 expression was significantly attenuated in Capan-1 relative to the other two cell lines (Figure 6d).

H3K27me3-derived nucleosome signal profiles at these promoters were concordant with the expression differences observed across samples. At the BCAT1 locus (Figure 6a), H3K27me3-associated nucleosome signal was stronger and more clearly phased in HPNE and PANC-1 than in Capan-1, with well-phased arrays detected near the TSS in both samples. A reciprocal pattern was observed at the CHD5 locus (Figure 6c), where Capan-1 displayed more abundant and better phased nucleosome signal, with a well-phased array identified near the TSS exclusively in this metastatic cell line. Within and flanking these arrays, motif hits for top-ranked NuRepress predictions with established relevance to pancreatic cancer fell predominantly within nucleosome occupancy valleys. Specifically, at the BCAT1 promoter, motif hits for RARA [44] and LRF [39] were detected within and adjacent to the well-phased arrays in HPNE and PANC-1 (Figure 6a), whereas at the CHD5 promoter, motif hits for MNT, a repressive MYC network component implicated in metastasis [49], were identified at analogous positions proximal to the Capan-1 array (Figure 6c). Notably, both RARA and MNT were also identified as candidate repressors by diffTF (Figure S6), lending additional support to these findings. This pattern highlights the clinical potential of NuRepress in linking regulatory features to disease progression and therapeutic target discovery.

Overall, these findings suggested that NuRepress could delineate stage-specific repressor-associated nucleosome reorganization at key promoters, linking the emergence and dissolution of well-phased arrays to corresponding shifts in transcriptional output and to the spatial distribution of candidate repressor binding motifs. This capacity to integrate repressive chromatin structure, nucleosome phasing, and transcriptional dynamics within a unified inferential framework positioned NuRepress as a principled approach for interrogating the repressive regulatory landscape across diverse biological and pathological contexts.

## 4. Discussion

Transcriptional repression is a fundamental regulatory process whose mechanistic underpinnings remain incompletely understood, yet the contribution of chromatin structural context to this process may not have been fully incorporated into regulatory inference frameworks. NuRepress addresses this gap by treating well-phased nucleosome arrays within repressive chromatin domains as discrete structural units that encode both the identity of the repressive chromatin state and the organizational features through which trans-acting repressors may act. Rather than centering the analysis on chromatin accessibility, NuRepress integrates histone modification-derived nucleosome signals with accessibility profiles, sequence features, and transcriptional outcomes to connect array identification, structural and functional stratification, and repressor prioritization within a unified analytical scheme.

The filtering of well-phased arrays on the basis of nucleosome signal strength, spacing regularity, and positional fuzziness can be understood as the selective detection of highly organized structural objects whose emergence may more plausibly reflect active regulatory processes rather than stochastic chromatin fluctuations [55,56,57]. The high degree of nucleosome ordering observed here is better explained by regulatory constraint than by complete functional neutrality. The differential positional preferences and accessibility profiles of distinct array subtypes may likewise be consistent with regulatory processes operating at different organizational scales. Building on this structural foundation, NuRepress incorporates genomic functional annotation and sample-level expression information to more finely partition the regulatory scope and repressive efficacy of each subtype, thereby strengthening the functional grounding of downstream repressor predictions.

TF binding and the local nucleosome reorganization it mediates represent one of the principal mechanisms through which nucleosome array phasing is established [58,59,60]. Grounded in this mechanistic rationale, NuRepress systematically characterizes the association between TF-binding signals within and flanking array boundaries—resolved across multiple distance scales—and gene expression downregulation, from which candidate repressors are prioritized. To provide an integrated assessment of binding potential and subtype-level repressive efficacy, NuRepress operationalizes this association through the RPS metric, enhancing both within-sample comparability and the biological interpretability of predictions.

When applied to the analysis of key gene regulatory changes during pancreatic cancer progression, NuRepress predictions showed considerable concordance with prior literature. Using repressive domain nucleosome phasing as the analytical entry point, NuRepress identified candidate repressors including RARA, MNT, and LRF, and implicated their potential regulatory involvement at loci including BCAT1 and CHD5—canonical pro-tumorigenic and tumor-suppressive genes in pancreatic cancer—thereby providing structurally contextualized evidence to support mechanistic interpretation of clinicopathological phenotypes and the identification of potential therapeutic targets. Notably, a subset of these candidates, including RARA and MNT, were also recovered by diffTF, offering complementary support from a distinct analytical framework. This is also compatible with current views of pancreatic cancer as a disease in which repressive chromatin dysregulation is functionally important, including H3K27me3-associated gene silencing and chromatin-remodeling defects that can perturb nucleosome positioning and transcriptional control [61].

More broadly, these findings highlight the potential clinical relevance of NuRepress as a framework for connecting repressive chromatin architecture with disease-associated transcriptional programs. By prioritizing candidate repressors in a structurally and functionally interpretable manner, NuRepress may support the discovery of clinically informative regulatory features and provide a complementary basis for future biomarker evaluation and mechanism-guided therapeutic exploration. Such applications may be of particular relevance in pancreatic cancer, where aberrant repressive chromatin organization is increasingly recognized as a contributor to pathological gene regulation, and epigenetic mechanisms are receiving growing translational attention [62,63,64].

Several aspects of NuRepress warrant further refinement. Although NuRepress does not impose constraints on the type of repressive histone modification supplied as input, different modification types carry inherently distinct signal distribution characteristics, genomic coverage patterns, and mechanistic associations. Accordingly, the current default parameter settings, which were established using H3K27me3-associated domains as the initial reference context, may not transfer optimally to other repressive marks without further adjustment. Systematic exploration of modification-aware parameterization, as well as more modification-aware strategies for repressive efficacy estimation and motif-based repressor identification, represent important directions for future development. In parallel, the current framework characterizes repressive regulation primarily through the integration of nucleosome array structural features, local sequence information, and expression downregulation signals. In biological reality, however, repressor activity is frequently coupled to co-repressor complex recruitment, local epigenetic state remodeling, and the active maintenance of silenced chromatin—processes not yet explicitly modeled within NuRepress. Incorporating broader multi-omics inputs and iterating predictions against experimental validation will therefore be important directions for improving the resolution, robustness, and mechanistic depth of repressor inference across diverse biological contexts.

Taken together, these considerations point to several meaningful directions for future refinement. Nevertheless, by integrating repressive chromatin context, phased nucleosome architecture, and transcription-associated signals within a unified analytical scheme, NuRepress already provides a structure-aware and mechanistically informative framework for repressor inference. This analytical foundation positions NuRepress for continued extension toward broader repressive chromatin contexts, richer regulatory modeling, and deeper biological interpretation.

## 5. Conclusions

NuRepress presents a computational framework for transcriptional repressor inference by exploiting phased nucleosome architecture within repressive chromatin domains. By integrating chromatin structure, local accessibility patterns, spatial motif information, and transcriptional output, it establishes an interpretable and structure-informed strategy for dissecting repressive regulation beyond accessibility-centered paradigms. The accessibility-based subtyping scheme identified by NuRepress showed reproducible organizational patterns across independent samples and across species, suggesting that the structural principles captured by the framework may reflect broadly shared features of repressive chromatin regulation. In the context of pancreatic cancer progression, NuRepress further identified candidate repressors with documented functional relevance and potential disease associations, supporting its utility as a general framework for investigating repressive regulatory mechanisms across diverse biological and pathological settings.

## Figures and Tables

**Table 1:** Repressive efficacy (RE) of different array subtypes across samples.

	HPNE		PANC-1		Capan-1	
	C1	C2	C1	C2	C1	C2
RE	3.83	57.80	5.53	62.40	5.02	45.71

## Data Availability

NuRepress is available at https://github.com/qianming-bioinfo/NuRepress (accessed on 14 April 2026).

## Supplementary Materials

The following supporting information can be downloaded at: https://www.mdpi.com/article/10.3390/genes17040480/s1, Figure S1: Comparison of sequence-related features across array subtypes; Figure S2: Cross-species validation of NuRepress accessibility-based subtyping in mouse Patski cells; Figure S3: Position-resolved subtype-relative motif enrichment around well-phased nucleosome array boundaries for CTCF, BORIS, and REST/NRSF; Figure S4: Mean-centered ATAC-seq signal profiles of the two array subtypes identified from H3K27me3 domains without nucleosome array phasing filtering across PDAC-related samples. Figure S5: Top-ranked candidate repressors are shown for the two inferred subtypes (C1 and C2) derived from H3K27me3 domains in PDAC-related samples; Figure S6: Heatmap of all candidate repressors identified by diffTF across HPNE, PANC-1, and Capan-1 comparisons; Table S1: Sample sizes and adjusted *p*-values for pairwise comparisons of array subtypes (C1_only, C2_only) vs. absent reference across HPNE, PANC-1, and Capan-1.
