# Supplementary material for: NuRepress: Inferring Transcriptional Repressors from Phased Nucleosome Architecture
Source: Genes (Basel). 2026 Apr 18;17(4):480. doi: 10.3390/genes17040480 (PMC13115820; doi:10.3390/genes17040480)
Supplement: Supplementary file 1 [file genes-17-00480-s001.zip › genes-4236154-supplementary.pdf]

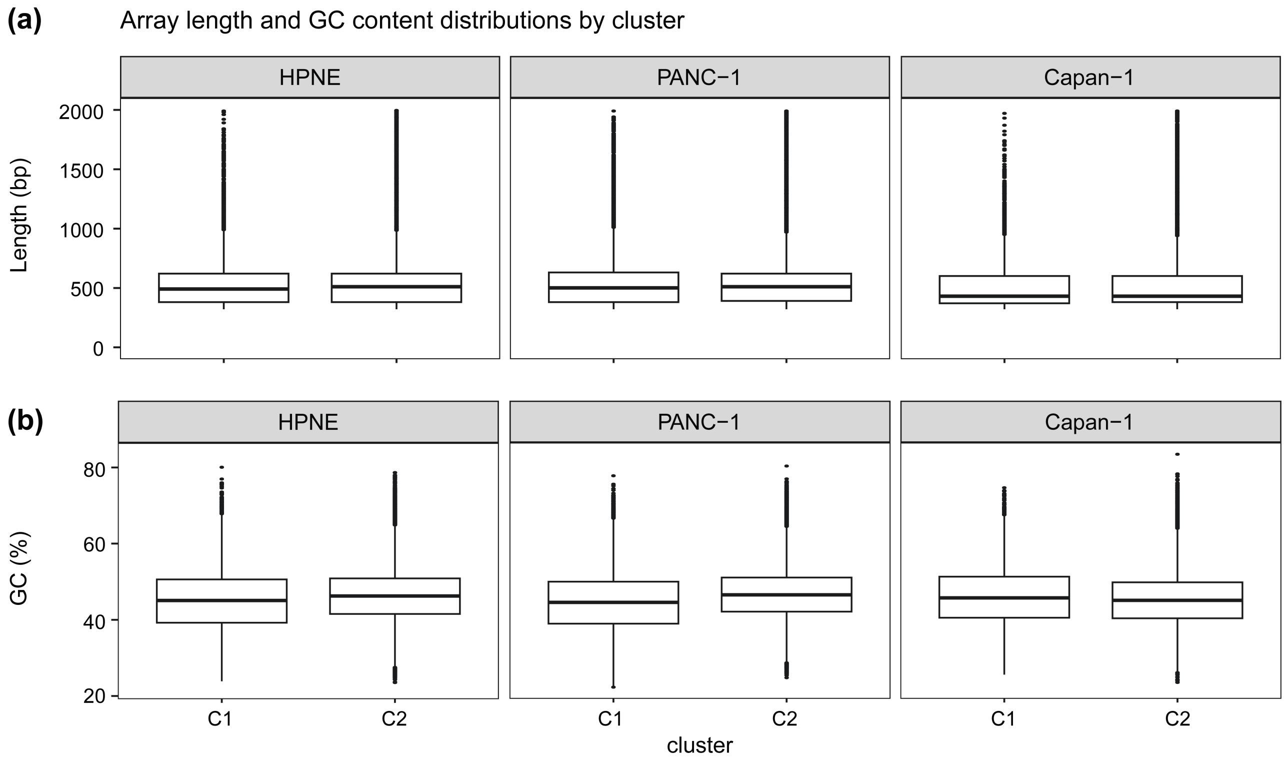

Figure S1. Comparison of sequence-related features across array subtypes. (a) Distribution of array lengths for C1 and C2 subtypes within each sample (HPNE, PANC-1, and Capan-1). (b) Distribution of GC content for C1 and C2 subtypes within each sample.

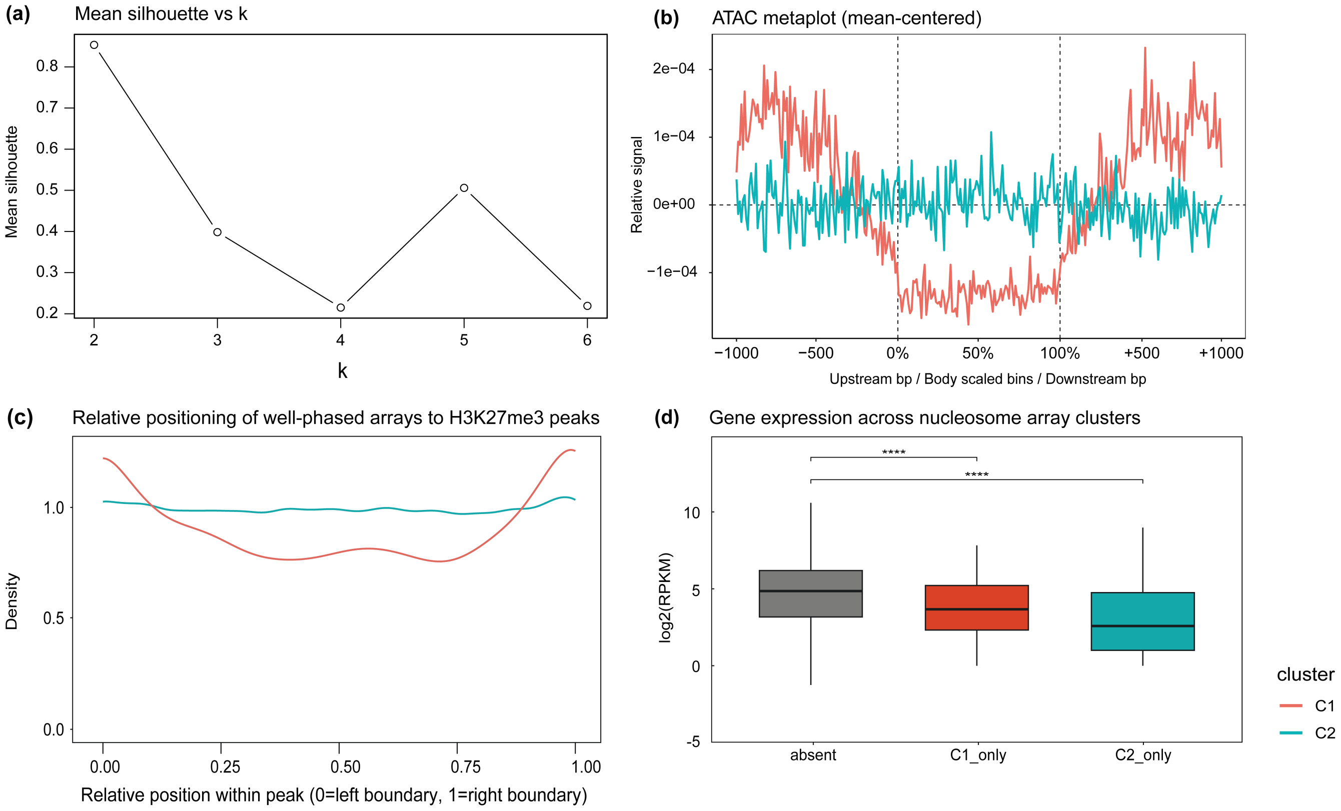

Figure S2. Cross-species validation of NuRepress accessibility-based subtyping in mouse Patski cells. (**a**) Mean silhouette scores across different values of *k*, indicating $k = 2$ as the optimal clustering solution. (**b**) Mean-centered ATAC-seq signal profiles for the two array subtypes, showing distinct accessibility patterns consistent with those observed in human samples. (**c**) Relative positioning of well-phased arrays within H3K27me3-enriched domains, demonstrating subtype-specific spatial distributions comparable to human data. (**d**) Gene expression levels across nucleosome array clusters, showing differential downregulation patterns between subtypes consistent with those observed in human samples. Asterisks denote BH-adjusted significance levels from pairwise Wilcoxon tests: * p.adj <= 0.05, ** p.adj <= 0.01, *** p.adj <= 0.001, and **** p.adj <= 0.0001.

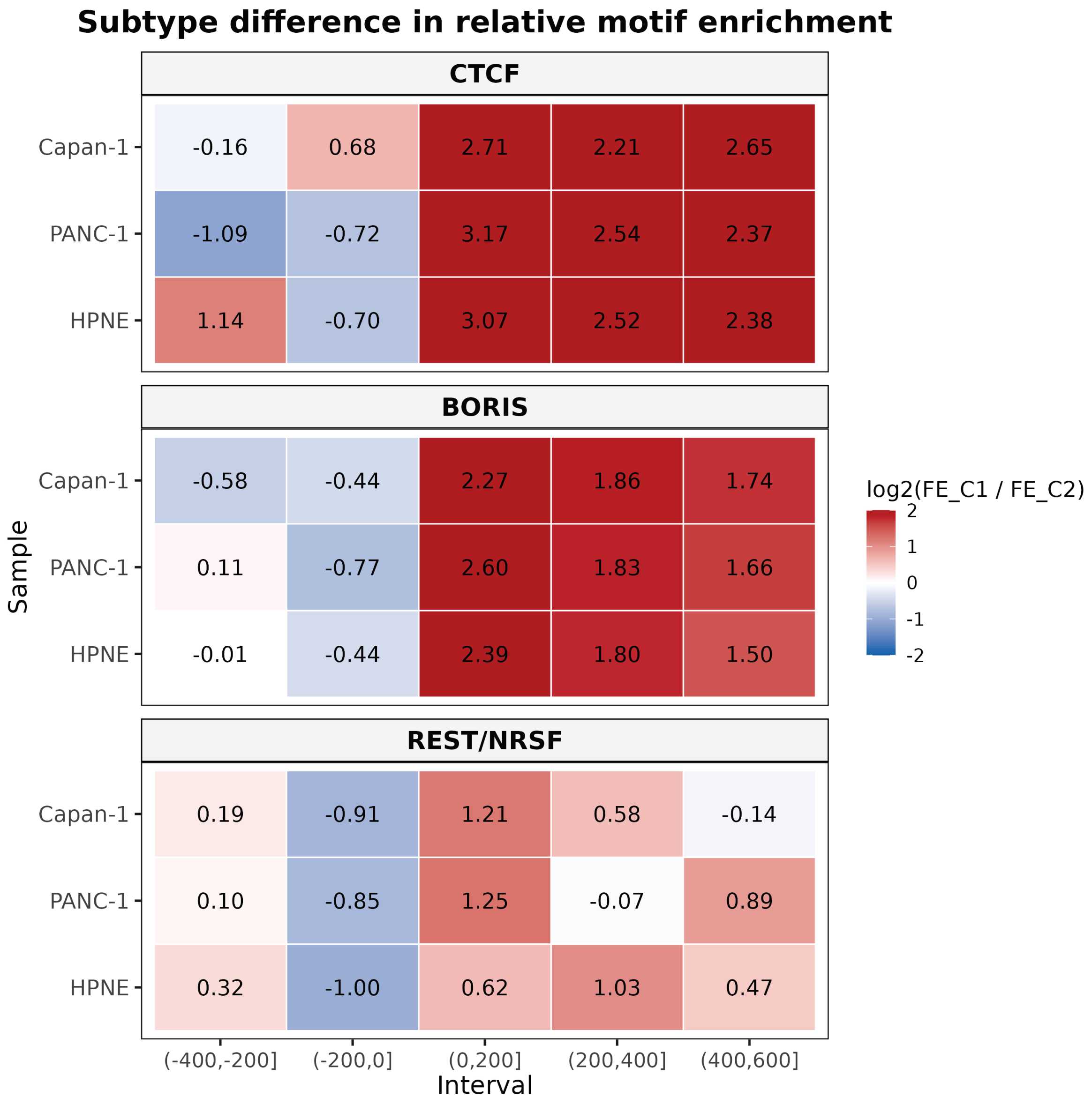

Figure S3. Position-resolved subtype-relative motif enrichment around well-phased nucleosome array boundaries for CTCF, BORIS, and REST/NRSF. Heatmaps show the relative motif enrichment difference between the two array subtypes (C1 and C2) across successive fixed 200-bp intervals around well-phased nucleosome array boundaries in HPNE, PANC-1, and Capan-1. For each motif, **FE (fold enrichment)** was defined as the ratio of the percentage of target sequences containing the motif to the percentage of background sequences containing the motif. The plotted value, **log2(FE_C1 / FE_C2)**, summarizes the subtype-relative enrichment bias within the same interval, with positive values indicating stronger enrichment in C1 and negative values indicating stronger enrichment in C2. Intervals are arranged from the inner side of the array boundary toward the outer side. Overall, CTCF and BORIS show a more consistent C1-biased enrichment pattern across the outer intervals, whereas REST/NRSF exhibits a more local and less spatially continuous subtype bias.

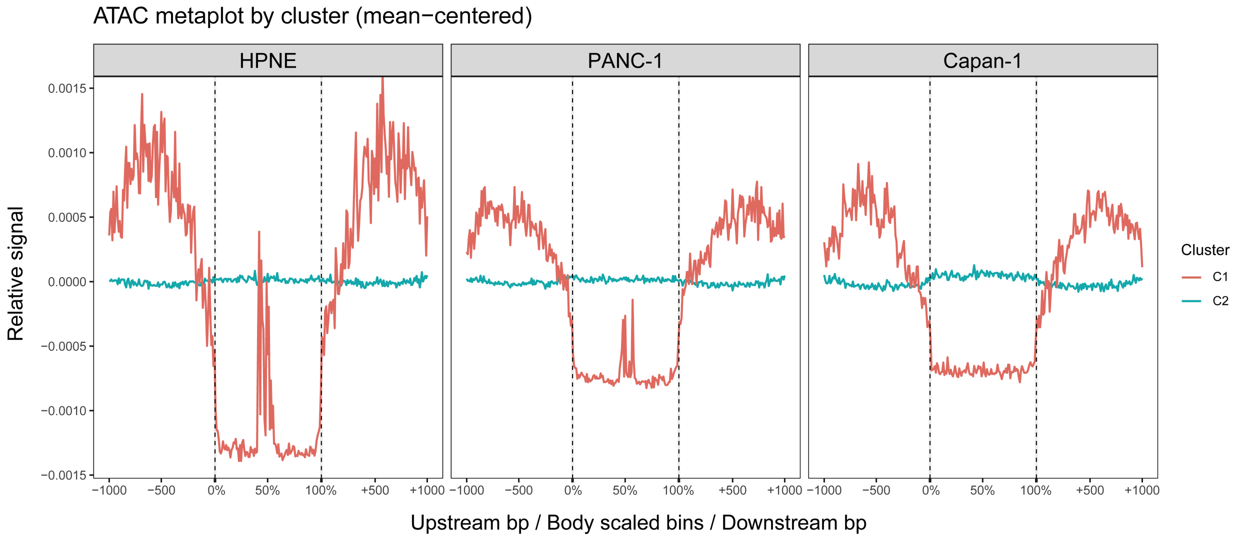

Figure S4. Mean-centered ATAC-seq signal profiles of the two array subtypes identified from H3K27me3 domains without nucleosome array phasing filtering across PDAC-related samples.

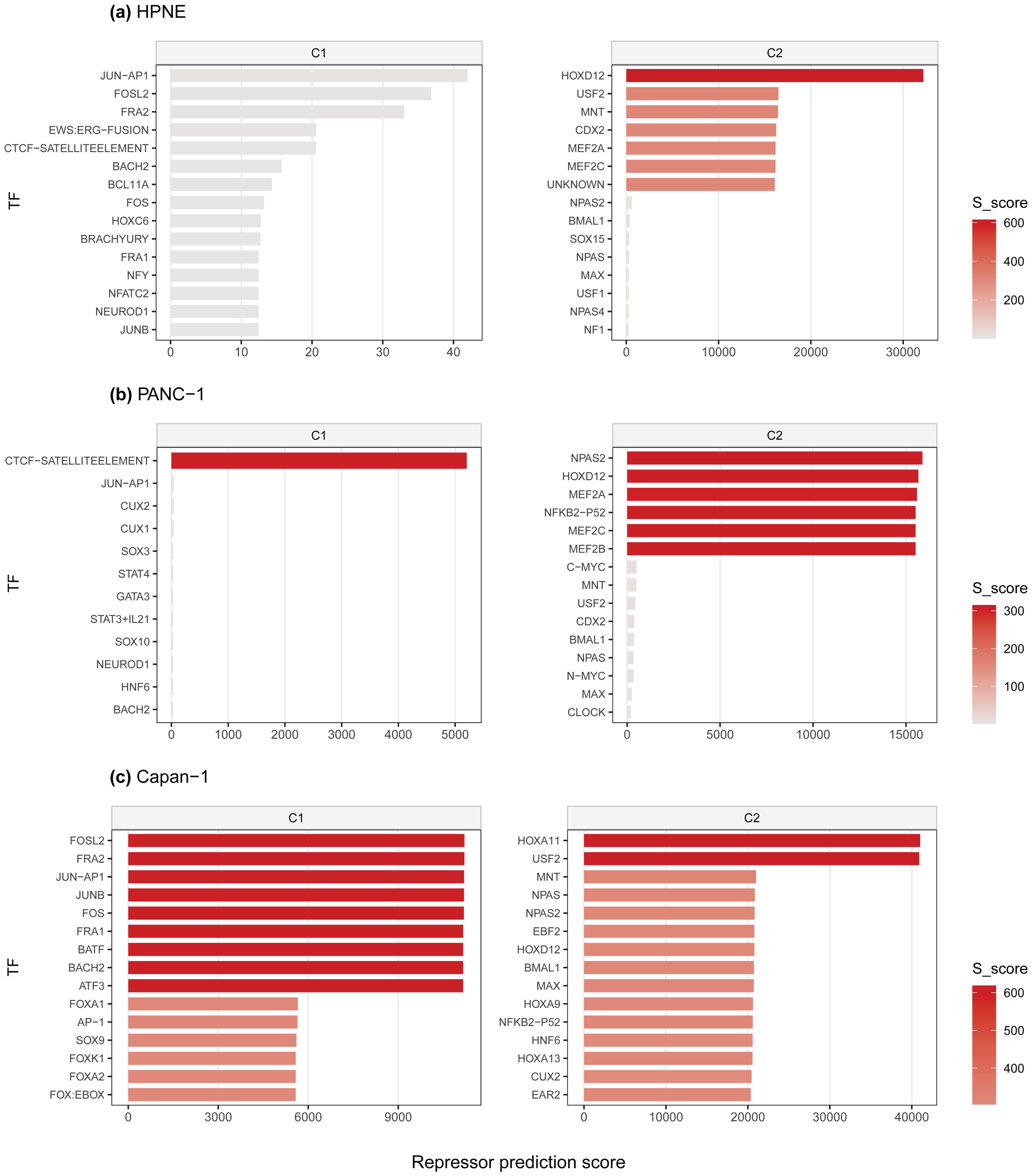

Figure S5. Top-ranked candidate repressors are shown for the two inferred subtypes (C1 and C2) derived from H3K27me3 domains in HPNE (a), PANC-1 (b), and Capan-1 (c) without applying nucleosome array phasing filtering. Bar length denotes the repressor prediction score (RPS), and bar color represents the corresponding subtype specificity score (S_score).

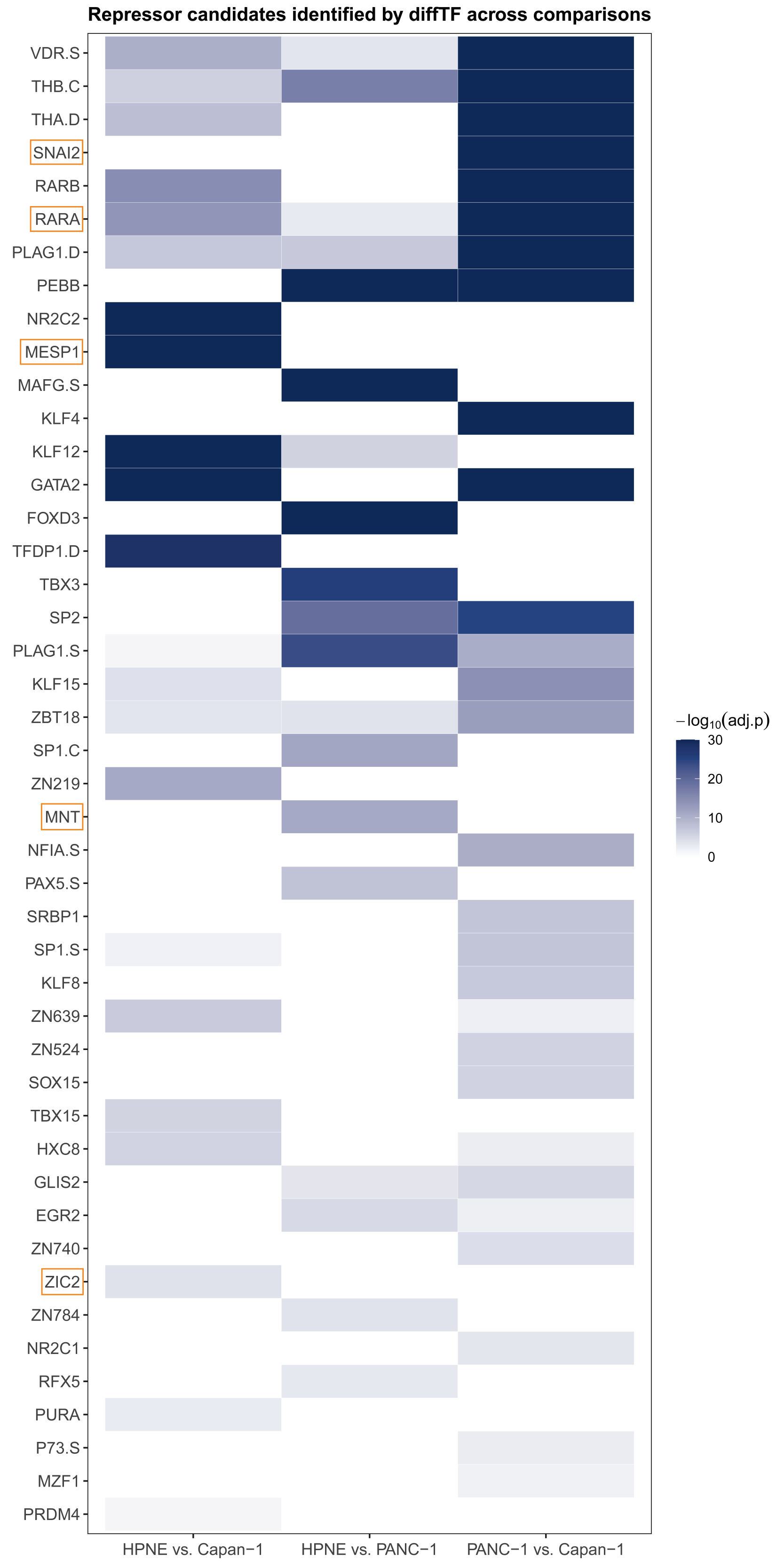

Figure S6. Heatmap of all candidate repressors identified by diffTF across HPNE, PANC-1, and Capan-1 comparisons (45 in total; adjusted p-value < 0.05 in at least one comparison). Orange boxes mark candidates that were also among the top 15 repressors predicted by NuRepress across the three samples (Figure 5). Blank cells indicate that the corresponding TF was not identified as a repressor in that comparison.

Table S1. Sample sizes and adjusted p-values for pairwise comparisons of array subtypes (C1_only, C2_only) vs. absent reference across HPNE, PANC-1, and Capan-1.

Sample	Comparison	n (Group1)	n (Group2)	Adjusted *p*-Value
HPNE	C1_only vs. absent	451	23,755	1.54×10^{-11}
HPNE	C2_only vs. C1_only	2648	451	4.04×10^{-26}
HPNE	C2_only vs. absent	2648	23,755	0 *
PANC-1	C1_only vs. absent	537	23,660	7.04×10^{-19}
PANC-1	C2_only vs. C1_only	2522	537	1.34×10^{-22}
PANC-1	C2_only vs. absent	2522	23,660	0 *
Capan-1	C1_only vs. absent	215	27,822	7.08×10^{-18}
Capan-1	C2_only vs. C1_only	1478	215	1.62×10^{-8}
Capan-1	C2_only vs. absent	1478	27,822	7.72×10^{-271}

Note: A value shown as 0 * indicates a statistically significant value smaller than the display precision.
